# Post-surgical External Coronary Artery Compression: A Rare Cause of ST Elevation Myocardial Infarction

**DOI:** 10.7759/cureus.39075

**Published:** 2023-05-16

**Authors:** Md Mashiul Alam, Michael Azrin, Yasir Adeel

**Affiliations:** 1 Internal Medicine, Bridgeport Hospital, Bridgeport, USA; 2 Interventional Cardiology, University of Connecticut Health Center, Farmington, USA; 3 Cardiology, University of Connecticut Health Center, Farmington, USA

**Keywords:** st elevation myocardial infarction (stemi), iatrogenic myocardial infarction, coronary artery occlusion, surgical aortic valve replacement (savr), chest tube

## Abstract

Iatrogenic ST elevation myocardial infarction (STEMI) after aortic valve surgery is a rare complication. Myocardial infarction (MI) due to mediastinal drain tube compression on the native coronary artery is also seen rarely. We present a case of ST elevation inferior myocardial infarction due to post-surgical drain tube placed after aortic valve replacement compressing on the right-sided posterior descending artery (rPDA).

A 75-year-old female presented with exertional chest pain and was found to have severe aortic stenosis (AS). After a normal coronary angiogram and proper risk stratification, the patient underwent surgical aortic valve replacement (SAVR). One day after surgery in the post-operative area, the patient was complaining about central chest pain suggestive of anginal pain. Electrocardiogram (ECG) revealed that she has ST elevation myocardial infarction in the inferior wall. Immediately, she was taken to the cardiac catheterization laboratory, which revealed that she has occlusion of the posterior descending artery due to compression by a post-operative mediastinal chest tube. All features of myocardial infarction resolved after simple manipulation of the drain tube.

The compression of the epicardial coronary artery after aortic valve surgery is very unusual. There are a few cases of other coronary artery compression due to mediastinal chest tube, but posterior descending artery compression causing ST elevation inferior myocardial compression is unique. Though rare, we need to be vigilant about mediastinal chest tube compression, which can cause ST elevation myocardial infarction after cardiac surgery.

## Introduction

The iatrogenic compression of the native epicardial artery is a rare phenomenon. There are a few cases of bypass graft [1-4] or native coronary artery [5,6] occlusion after cardiac surgery by a mediastinal drain tube. We present a case of right-sided posterior descending artery (rPDA) compression after surgical aortic valve replacement (SAVR) due to a misplaced mediastinal chest tube causing ST elevation myocardial infarction (STEMI). STEMI was resolved after the chest drain was pulled with a simple maneuver.

## Case presentation

A 74-year-old female with no past medical illness presented with exertional chest pain and was found to have severe aortic stenosis (AS). Electrocardiogram (ECG) shows features of left ventricular hypertrophy (LVH). On echocardiography, her mean pressure gradient across the aortic valve was 47 mmHg. As she had symptomatic severe aortic stenosis (AS) without any other significant comorbidities, the plan was made to do surgical aortic valve replacement (SAVR). She underwent preoperative left heart catheterization, which shows normal epicardial coronary arteries.

Eventually, the patient underwent a surgical replacement of the stenosed aortic valve with a bioprosthetic valve. The surgery was uneventful, and the patient was sent to the cardiac ICU for monitoring. A mediastinal drain tube was put in to prevent fluid accumulation after cardiac surgery. On post-operative day 1, the patient was complaining of central chest pain with radiation to the arm. ECG was done showing ST elevation in leads II, III, and arteriovenous fistula (aVF) with baseline LVH. It was puzzling as previously, she had no ECG changes suggestive of ischemic heart disease (IHD), and left heart catheterization was also normal. Bedside echocardiogram revealed wall motion abnormality in the middle and distal inferior wall with mildly reduced LV function. STEMI alert was activated, and the patient was taken to the catheterization laboratory emergently.

**Figure 1:**
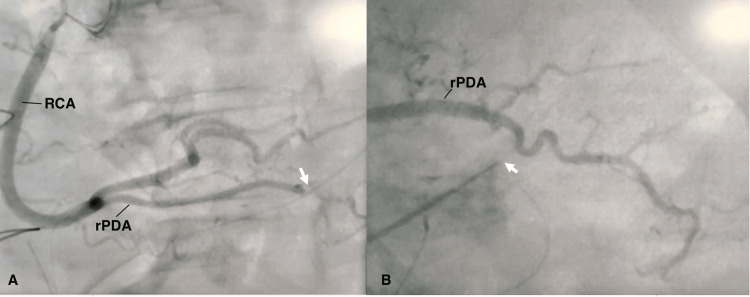
Mediastinal drain tube compressing the coronary artery (A) Mediastinal drain tube compressing on the right PDA (white arrow pointing to the site of compression). (B) Normal flow in the rPDA after the drain tube was pulled (white arrow showing the tip of the drain tube) RCA, right coronary artery; rPDA, right-sided posterior descending artery; PDA, posterior descending artery

A coronary angiogram revealed that the chest tube is intermittently compressing on the right-sided posterior descending artery (rPDA) limiting blood flow distal to the compression (Figure 1A). The drain tube was pulled until there was no compression on the rPDA and there was a restoration of thrombolysis in myocardial infarction (TIMI) 3 flow (Figure 1B). The patient’s symptoms of chest pain were also relieved after that with the normalization of ECG and echocardiographic findings. The rest of the hospital course was uneventful, and she was discharged home after usual post-operative care.

## Discussion

We present a case of posterior descending artery compression by a mediastinal drain tube placed after SAVR, which is the first reported case. In the past, there were a few reports of native coronary artery or graft compression.

Most complications of chest tube placement come from closed pleural chest tube placement, which could be benign to serious complications [7]. There have been reports of unusual complications from close chest tube insertion such as injuries to the lung, perforation of the diaphragm or abdominal organs, cardiac injuries, and common complications such as pneumothorax or subcutaneous emphysema [7,8].

Complications from open mediastinal tube placement after cardiac surgery are usually benign and underreported. Pneumothorax after removing such tubes or patients suffering from pain due to the tube placement is most encountered by cardiac surgeons [4]. Yet, there are reports of serious complications such as intestinal perforation or vein graft rupture causing cardiogenic shock [4,9]. There are reports of saphenous vein graft [1,2,4] or internal mammary artery [3] obstruction causing cardiogenic shock. The compression of native coronary arteries can also cause cardiogenic shock [6] or post-operative myocardial infarction (MI) [5].

After surgical aortic valve replacement (SAVR), myocardial ischemia or infarction could be due to supply-demand mismatch as a result of aortic cross-clamping causing ischemia in hypertrophied left ventricle (LV) [10]. STEMI after surgical aortic valve surgery is usually due to localized trauma to the native artery, embolization, or spasm [10], which is uncommon. Our case is unique in this regard as there is no mention of STEMI due to the compression of rPDA by a drain tube.

## Conclusions

Being vigilant about post-surgical drain tubes that may cause compression in the coronary artery can be an easy fix and prevent dreaded complications. Mediastinal chest tubes causing the occlusion of the native coronary arteries are unexpected and easily overlooked.

## References

[1] Heestermans TM, Dambrink JH, Sie HT (2009). Immediate myocardial infarction due to compression of a vein graft. Ann Thorac Surg.

[2] Rahim MA, Petrossian G, Edens M, Abittan N, Rahim H, Ali ZA (2021). Cardiogenic shock secondary to mediastinal tube compression of a saphenous vein graft. Coron Artery Dis.

[3] Knipp S, Massoudy P, Piotrowski JA, Jakob H (2002). Pitfall in coronary artery bypass surgery: poor flow of left internal mammary artery to left anterior descending artery graft due to compression by a chest drain. Eur J Cardiothorac Surg.

[4] Svedjeholm R, Håkanson E (1997). Postoperative myocardial ischemia caused by chest tube compression of vein graft. Ann Thorac Surg.

[5] Sulimovic S, Noyez L (2006). Postoperative myocardial ischemia caused by compression of a coronary artery by chest tube. J Cardiovasc Surg.

[6] Langer NB, Nazif TM, Powers ME, Fukuhara S, Borger MA, George I (2016). Cardiogenic shock from coronary compression: a difficult diagnosis but easy fix. Ann Thorac Surg.

[7] Miller KS, Sahn SA (1987). Chest tubes. Indications, technique, management and complications. Chest.

[8] Berkow AE, Salo BC (1977). The kinky chest tube: a sign of entrapment following median sternotomy. AJR Am J Roentgenol.

[9] Kollef MH, Dothager DW (1991). Reversible cardiogenic shock due to chest tube compression of the right ventricle. Chest.

[10] Sharratt GP, Rees P, Conway N (1976). Myocardial infarction complicating aortic valve replacement. J Thorac Cardiovasc Surg.

